# Annotations

**Published:** 1955-11

**Authors:** 


### *Annotations*

### HEALTH CLINICS IN BRISTOL

Under the inspiring guidance of Dr. R. H. Parry, Medical Officer of Health, Bristol has had for many years a proud and impressive record for providing health clinics. Many of our readers will be familiar with, indeed probably will have worked in, the pre-war buildings at Southmead, Tower Hill, Speedwell, Portway and Bedminster.

Some ten years ago, Dr. Parry produced a plan (accepted in principle by the Health Committee and City Council) providing for a network of clinics to serve all areas of the City and to ensure that no mother would have to travel further than half a mile to the nearest centre. Post-war developments of new housing estates on the periphery of the City have led to some modification of the overall plan but its main provisions are being implemented steadily.

In the post-war years new clinics have been established at Frenchay Hospital (jointly with the Hospital Management Committee), on the Lawrence Weston housing estate, at Brooklea (St. Anne's Park district), in Knowle (William Budd Health centre) and at Granby House (Bedminster district).

The Charlotte Keel clinic, a two-storied building of substantial proportions, is nearing completion. This clinic will serve the Lower Ashley Road—Stapleton Road area and it is hoped that the Minister of Health will be present at the official opening towards the end of the year.

The Hartcliffe estate will also soon have its own clinic for Ministry approval has now been given for building to start. Henbury and Brentry clinic has been approved in principle and building is likely to be started before the end of the year.

This programme will be of great interest to the many Bristol general medical practitioners who do their ante-natal work in the Health Department's clinics. There can be little doubt that this policy of providing first-class health services readily available to the people is contributing to Bristol's remarkably low infant mortality rate of 20 per 1,000 live births.

### RE-ARRANGEMENTS OF THE HEALTH VISITOR SERVICE FOR THE AGED AND CHRONIC SICK

Hitherto applications for the services of the specialized health visitor to help with the aged and chronic sick have been made to the Central Health Clinic. Owing to rapid expansion it has become necessary to de-centralize this work. As from 1st August, 1955, the city will be divided into four divisions for this purpose, each division being based at one of the main health clinics.

In future, applications relating to patients living in a particular area should be made to the nearest divisional health clinic as indicated below:

(1) *Central Clinic, Tower Hill, Bristol 2.* — Specialized health visitor: Sister Sheen, Tel. 2-6602. The Central Clinic will cover St. Jude's, St. James', St. Agnes', St. Paul's, St. Werburgh's, Baptist Mills, Hotwells, Clifton Wood, Montpelier, Lower Easton, Lower Whitehall, Barton Hill, Lawrence Hill, Newtown and Stapleton Road (below the traffic lights).

(2) *Southmead Clinic, Monks Park Avenue, Southmead.* — Clinic Sister: Sister E. Jones, Tel. 62-6414. Specialized health visitor: Sister Newns. This clinic will cover Avonmouth, Lawrence Weston, Henbury, Shirehampton, Sea Mills, Southmead, Horfield, Lockleaze, Bishopston, St. Andrews, Redland, Clifton and Cotham.

(3) *Speedwell Clinic, Whitefield Road, Bristol 5.* — Clinic Sister: Sister Humber, Tel. 7-3194. Specialized Health Visitor: Sister Aplin. This clinic will cover Brislington, St. Anne's, St. Anne's Park, Stapleton, Frenchay, Frenchay Park, Eastville, Upper Easton, Greenbank, St. George, Crews Hole, Redfield, Speedwell, Fishponds and St. Philip's Marsh.

(4) *Bedminster Clinic, Wedmore Vale, Bristol 3.* — Clinic Sister: Sister Tacker, Tel. 6-3798. Specialized health visitor: Sister Harris. This clinic will cover Totterdown, Knowle, Hengrove, Whitchurch, Knowle West, Hartcliffe, Withywood, Highridge, Bishopsworth, Bedminster Down, Bedminster, Southville, Ashton and Redcliff.

Telephone calls and written applications should be made to the appropriate Clinic Sister.

